# Life-Threatening Airway Obstruction Due to Endobronchial Blood Clot Following Endobronchial Ultrasound-Guided Transbronchial Needle Aspiration (EBUS-TBNA) in a Patient With Metastatic Renal Cell Carcinoma

**DOI:** 10.7759/cureus.97720

**Published:** 2025-11-25

**Authors:** Hafiz Sohail Kamran, Sana Aftab, Wai Sun Lam, Mazhar Chuadri, Asif Azam

**Affiliations:** 1 Respiratory Medicine, Russells Hall Hospital, Dudley, GBR

**Keywords:** ebus and interventional bronchoscopy, endotracheal intubation, flexible bronchoscope, naloxone, post-cpr

## Abstract

Endobronchial ultrasound-guided transbronchial needle aspiration (EBUS-TBNA) is a widely used minimally invasive procedure for mediastinal lymph node sampling. Complications are infrequent, and life-threatening airway obstruction due to post-procedural intrabronchial blood clot formation is extremely rare. We report the case of a 76-year-old woman with metastatic clear cell renal cell carcinoma (RCC) who went into cardiorespiratory arrest immediately after the procedure. Bronchoscopic removal of a large blood clot, which had occluded the left main bronchus, resulted in rapid clinical improvement. This case highlights the importance of prompt recognition and multidisciplinary management of this rare but critical complication.

## Introduction

Endobronchial ultrasound-guided transbronchial needle aspiration (EBUS-TBNA) is a procedure that uses a flexible bronchoscope with an ultrasound probe to examine the airways and nearby structures in the lungs. It helps determine whether an abnormal area is cancerous, assess the size of a cancer, and check if it has spread to other areas or lymph nodes. During the procedure, the doctor can view abnormalities and take tissue samples (biopsies) for testing. EBUS-TBNA is a well-established procedure for mediastinal staging and diagnosis of thoracic malignancies, offering high diagnostic yield with a favourable safety profile [1].

Common complications are generally minor, such as transient haemoptysis, but severe bleeding leading to airway obstruction is exceedingly uncommon [2]. Patients with highly vascular tumours, such as renal cell carcinoma (RCC), may be at increased risk of bleeding complications during biopsy procedures. We present a rare case of airway obstruction caused by an intrabronchial blood clot immediately following EBUS-TBNA in a patient with metastatic RCC. Other complications of EBUS include cough, infection, pneumothorax, complications associated with sedations, and sore throat or hoarseness.

## Case presentation

A 76-year-old woman with a history of metastatic clear cell RCC and bilateral pulmonary metastases diagnosed 18 months previously was referred for evaluation of an enlarging subcarinal lymph node noted on routine surveillance CT. The differential diagnosis was progression of metastatic disease and granulomatous inflammation. Her case was discussed in the Lung multidisciplinary team (MDT), and a decision was made to do EBUS and perform a tissue biopsy for further testing.

Her oncologic treatment entailed 18 cycles of first-line targeted therapy resulting in stable disease, followed by initiation and subsequent discontinuation of palliative immunotherapy due to intolerance, and resumption of targeted therapy with ongoing clinical stability.

**Figure 1:**
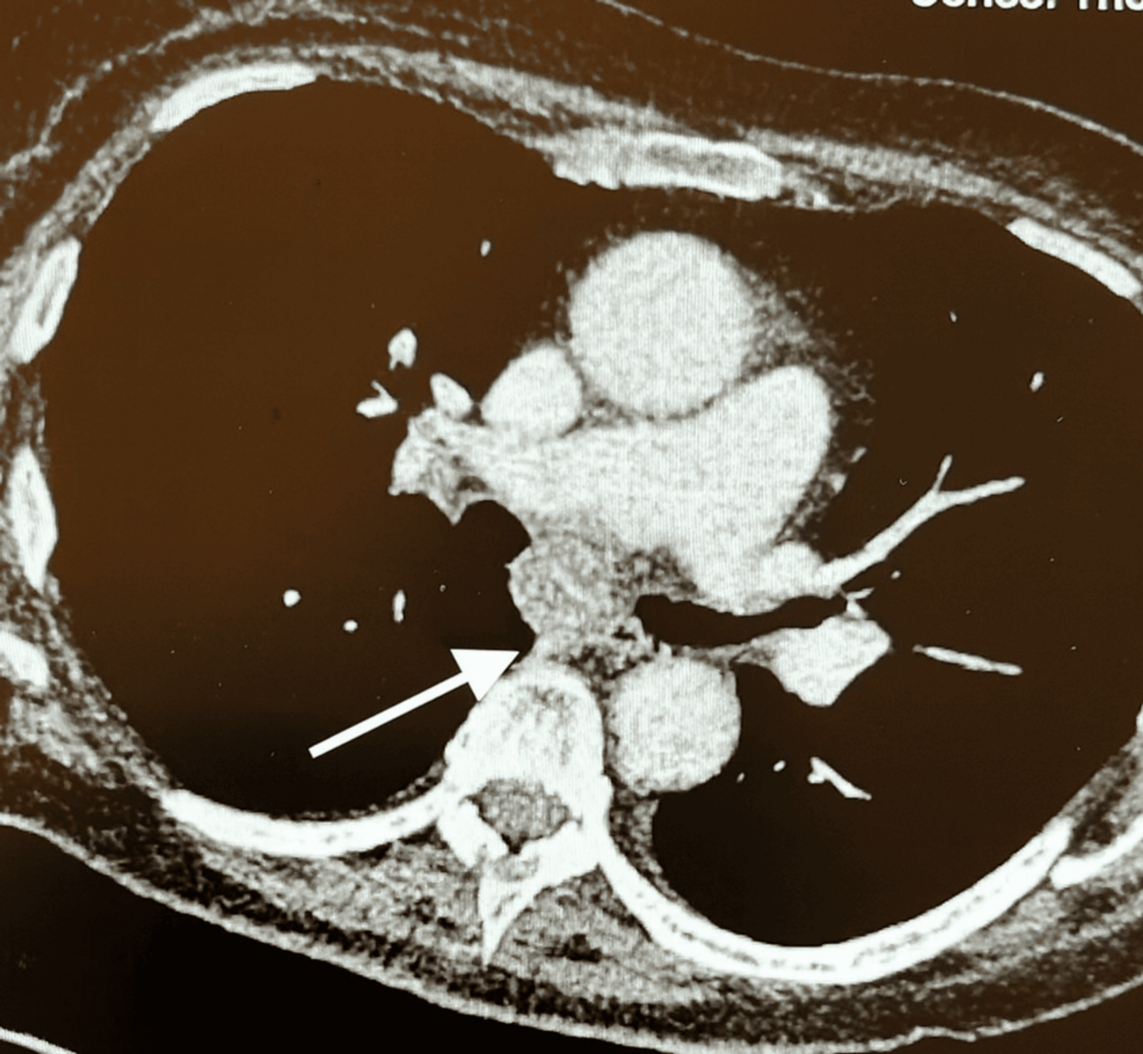
CT scan showing enlarged subcarinal lymph node (arrow)

CT scan (Figure 1) revealed no significant changes in bilateral lung metastases compared to previous scans, a right renal mass lesion of 74 mm, obstructing the proximal right ureter, causing mild hydronephrosis. A new enlarged subcarinal lymph node of 21 mm was noted. 

Pre-procedural evaluation revealed a normal international normalised ratio (INR) of 1.2 and no use of anticoagulants. Spirometry was normal: forced vital capacity (FVC) 2.36 L (89% predicted), forced expiratory volume in one second (FEV1) 1.70 L (84% predicted), and FEV1/FVC 72%. The patient underwent uneventful EBUS-TBNA under conscious sedation (intravenous midazolam 1 mg and fentanyl 75 mcg.

Immediately post extubation, her oxygen saturation plummeted to the 50s, progressing rapidly to respiratory and cardiac arrest. Following one cycle of cardiopulmonary resuscitation (CPR), spontaneous circulation was restored. Initial suspicion of sedation-induced hypoventilation prompted administration of naloxone, which improved alertness, but hypoxia persisted. Fresh haemoptysis was noted. We administered 1 g of intravenous tranexamic acid. On examination, she was noted to have diminished chest expansion and breath sounds on the left side.

**Figure 2:**
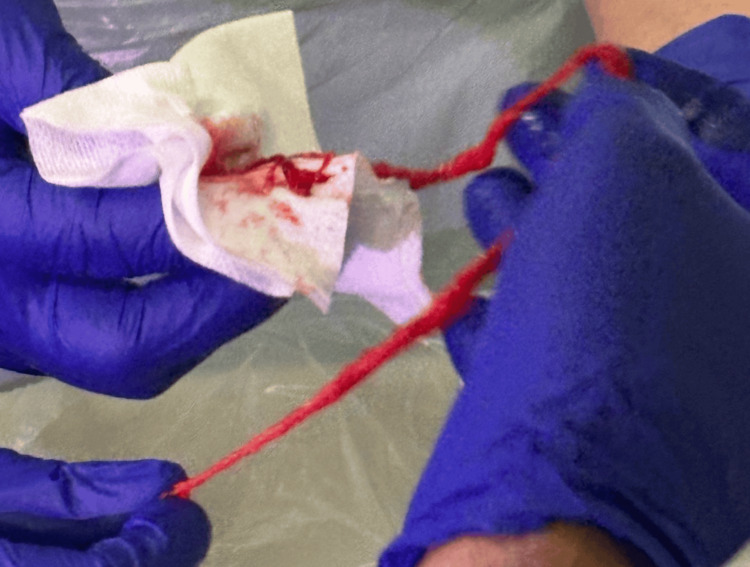
Endobronchial blood clot retrieved via flexible bronchoscopy

**Figure 3:**
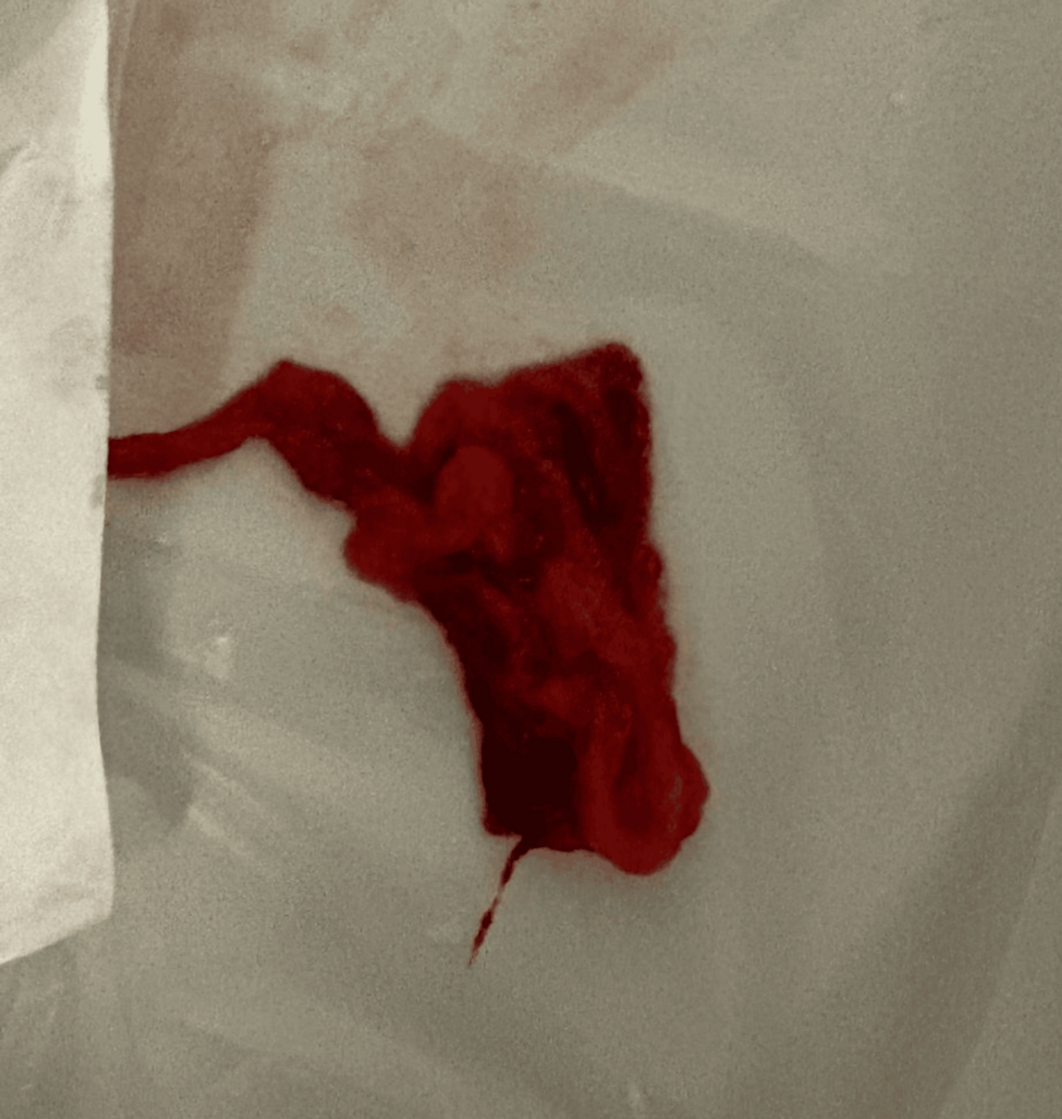
Endobronchial blood clot retrieved via flexible bronchoscopy that was obstructing the left main bronchus

Endotracheal intubation (8 mm tube) was performed; however, oxygenation remained stubbornly below 70% despite 100% FiO2 and maximal ventilator support. We were unable to pass the bronchoscope down the endotracheal tube (ETT). We elected to pass the bronchoscope through the nose. The ETT cuff was deflated, and the flexible bronchoscope was advanced alongside the ETT, revealing complete occlusion of the left main bronchus by a large blood clot. As Figures 2, 3 show, a large blood clot was retrieved from the left main bronchus. Attempts to break up the clot using boluses of 20 ml of normal saline were unsuccessful. We applied high suction and removed the bronchoscope along with a large, organised clot, resulting in immediate improvement in oxygenation.

The patient was transferred to intensive care and successfully extubated the following day without neurological sequelae. Histopathological analysis confirmed metastatic clear cell RCC in the sampled lymph node. There were fragments of blood clot with tumour cells arranged in discohesive sheets. Many of the cells had abundant eosinophilic cytoplasm and eccentrically placed round nuclei with prominent nucleoli, rendering a distinctly rhabdoid appearance. The nuclei exhibited moderate to marked pleomorphism. There were a few clusters of cells with moderate amounts of clear cytoplasm. These cells were positive for PAX8, vimentin, and CD10.CK7, CK20, CAIX, RCC, p40, and Napsin A were negative. TTF1 showed focal weak positivity, which was taken as negative. The morphology and immuno-phenotype were in keeping with a metastatic RCC.

## Discussion

This case illustrates a rare but serious complication of EBUS-TBNA: acute airway obstruction caused by intrabronchial blood clot formation. While minor haemoptysis is frequently observed post-EBUS and generally self-limiting, massive bleeding resulting in airway obstruction is exceptionally rare [3]. The hyper-vascular nature of RCC metastases likely contributed to the significant haemorrhage in this patient.

Published data suggest that patients with vascular tumours undergoing EBUS-TBNA may require enhanced procedural caution [4]. Pre-procedure assessment of bleeding risk and ensuring the immediate availability of bronchoscopic and airway management resources are critical. Furthermore, sedation and intubation strategies should facilitate rapid access to the airway in emergencies. Multidisciplinary collaboration among respiratory physicians, intensivists, and anaesthetists was pivotal in timely diagnosis and management, enabling a favourable outcome.

## Conclusions

Clinicians should recognise that significant airway obstruction due to blood clot formation is a rare but life-threatening complication following EBUS-TBNA, especially in patients with vascular malignancies such as RCC. Prompt bronchoscopy and multidisciplinary emergency airway management are crucial for optimising patient outcomes.
